# Application of robot-assisted endoscopic technique in the treatment of patent ductus arteriosus in 106 children

**DOI:** 10.1007/s11701-023-01537-7

**Published:** 2023-01-16

**Authors:** Liyang Ying, Xueke Wang, Xiwang Liu, Zheng Tan, Jiangen Yu, Lijun Yang, Qiang Shu

**Affiliations:** Department of Cardiac Surgery, The Children’s Hospital, Zhejiang University School of Medicine, National Clinical Research Center for Child Health, Hangzhou, China

**Keywords:** Robotic surgical technique, Patent ductus arteriosus, Faster recovery, Less trauma, Children

## Abstract

The objective is to evaluate and apply the robot-assisted endoscopic surgical technique for treatment of patent ductus arteriosus (PDA) in children. Clinical data of 106 children with PDA who underwent robot-assisted endoscopic operation were retrospectively analyzed from August, 2020 to March, 2022. Demographic and preoperative data were collected, including the patient’s age, weight, diameter of the ductus arteriosus, operation time, length of postoperative hospital stay, postoperative complications and hospitalization cost. The age ranged from 6 months to 12 years with median age of 2.5 years. In addition, the weight ranged from 6.6 kg (kg) to 51.6 kg with median weight of 12.5 kg. Patients who received transcatheter PDA closure were also enrolled during the same period. Clinical features and perioperative data were compared between the two groups. All the 106 cases underwent robotically assisted surgery for PDA ligation. No one was converted to thoracotomy. The length of operation time was 15–84 min, with an average of 39.4 min. There was no obvious bleeding during the operation. The length of postoperative hospital stays were 1–3 days, with an average of 1.1 ± 0.2 days, which was significantly shorter than that of patients underwent transcatheter approach PDA closure (2.2 ± 0.2 days) (*p* < 0.05). The average hospitalization costs were US$ 8180 in the 106 patients, which were more expensive than that of ones who received transcatheter procedure (US$ 5076 ± 406) (*p* < 0.05). Only one case was found to have residual ductus shunt during early postoperative follow-up. One case was found with recurrent laryngeal nerve injury. The two cases recovered after 3 months of follow-up. The median duration of follow-up was 12 (1–20) months. No other short-term complications occurred during the follow-up period. Robotic surgical technique for PDA ligation in children is a safe, effective and reliable surgical method with less trauma, faster recovery and fewer surgical risks. This approach should be considered as an option in children patients requiring PDA ligation.

## Introduction

Patent ductus arteriosus (PDA) is a common congenital cardiac anomaly, accounting for approximately 5–10% of congenital heart diseases [1]. Due to the left-to-right shunt, PDA leads to increase in continuous pulmonary blood flow and left ventricular preload. Without treatment, PDA could put patient at high risk for heart failure, infective endocardium, pulmonary hypertension and other health hazards which require surgical treatment [2, 3]. Traditionally, thoracotomy ligation, percutaneous puncture occlusion and thoracoscopic ligation had been performed to treat PDA. Robotic surgery was becoming a safe alternative to the traditional approaches in cardiac surgery after the advent of the robotic DaVinci system [4]. Robotic surgical technique in cardiac disease had been performed with favorable results; however, most patients were adults and selected adolescent. There were limited data about the use of robotics for younger children, in particular infants and young children. It had been reported, in a small number of patients, that robotic technology could be used for closure of PDA [5]. Nonetheless, there are still limited data on the adoption of robotics to PDA ligation and the current knowledge needs to be updated, especially in the younger children. In the present study, we present our experience of PDA ligation using the fourth-generation Da Vinci-assisted thoracoscopic technique, and tried to evaluate its feasibility and safety.

## Materials and methods

### Patients

Between August, 2020 and March, 2022, clinical data of 106 children who underwent robot-assisted thoracoscopy technique for PDA ligation in our center were retrospectively reviewed. Preoperative echocardiography showed that the diameter of PDA ranged from 0.15 cm to 0.71 cm and that there was persistent left-to-right shunt at the ductus with machinery murmur. Fifty-six patients suffered recurrent respiratory tract infections. Chest X-radiograph showed increased pulmonary blood and left ventricular enlargement in 50 cases. ECG showed high left ventricular voltage in 28 cases. CT angiography was performed in one case to exclude the constrictive descending aorta. Simultaneously, data of 225 patients received transcatheter PDA closure were also collected.

The included patients had stable clinical conditions for at least 1 month. Patients were excluded if they were premature with bronchopulmonary dysplasia, had abnormal liver or renal function, had major chromosomal abnormalities, exhibited pulmonary inflammation before the surgery, had severe pulmonary hypertension with right-to-left shunt at the ductus, or refused to participate in the study.

Before operation, all patients were informed of all treatment options for PDA, which including thoracotomy ligation, percutaneous puncture occlusion and robot-assisted thoracoscopy surgery. The patients and minor(s)’ legal guardian/next of kin chose one of the surgical procedures and signed an informed consent. This choice was entirely in accordance with their wishes, because all methods could be used to treat these patients. This study protocol was approved by the Ethics Committee of Children’s Hospital Zhejiang University School of Medicine.

### Study protocol

The study was performed using standard protocols as shown in following. Surgery was performed under general anesthesia with endotracheal intubation. The tracheal intubation was inserted into the right mainstem bronchus for one-lung ventilation. The arterial blood pressure monitoring and central catheterization of the internal jugular vein were established. All patients underwent routine hemodynamic and blood gas surveillance. Routine monitoring also included transcutaneous oxygen saturation, continuous end-tidal carbon dioxide, blood pressure, and an electrocardiogram.

Patients were routinely extubated in the operating room and were transported to the general ward. Echocardiography and chest X-ray were routinely reviewed the next morning. Patients were discharged on postoperative day 1 or 2 in the absence of complications. Patients were regularly followed up at 1, 3 and 6 month after discharge. Follow-up data were obtained through clinic visits. Follow-up results included all complication data.

Patients who chose to receive transcatheter PDA closure were also enrolled during the same period. Generally, in our center, the occluder device shall be confirmed to be free of displacement by echocardiography and chest X-ray on postoperative Day 2 or 3 before discharge. Clinical features and perioperative data were compared between the two groups.

### Surgical procedure

**Fig. 1:**
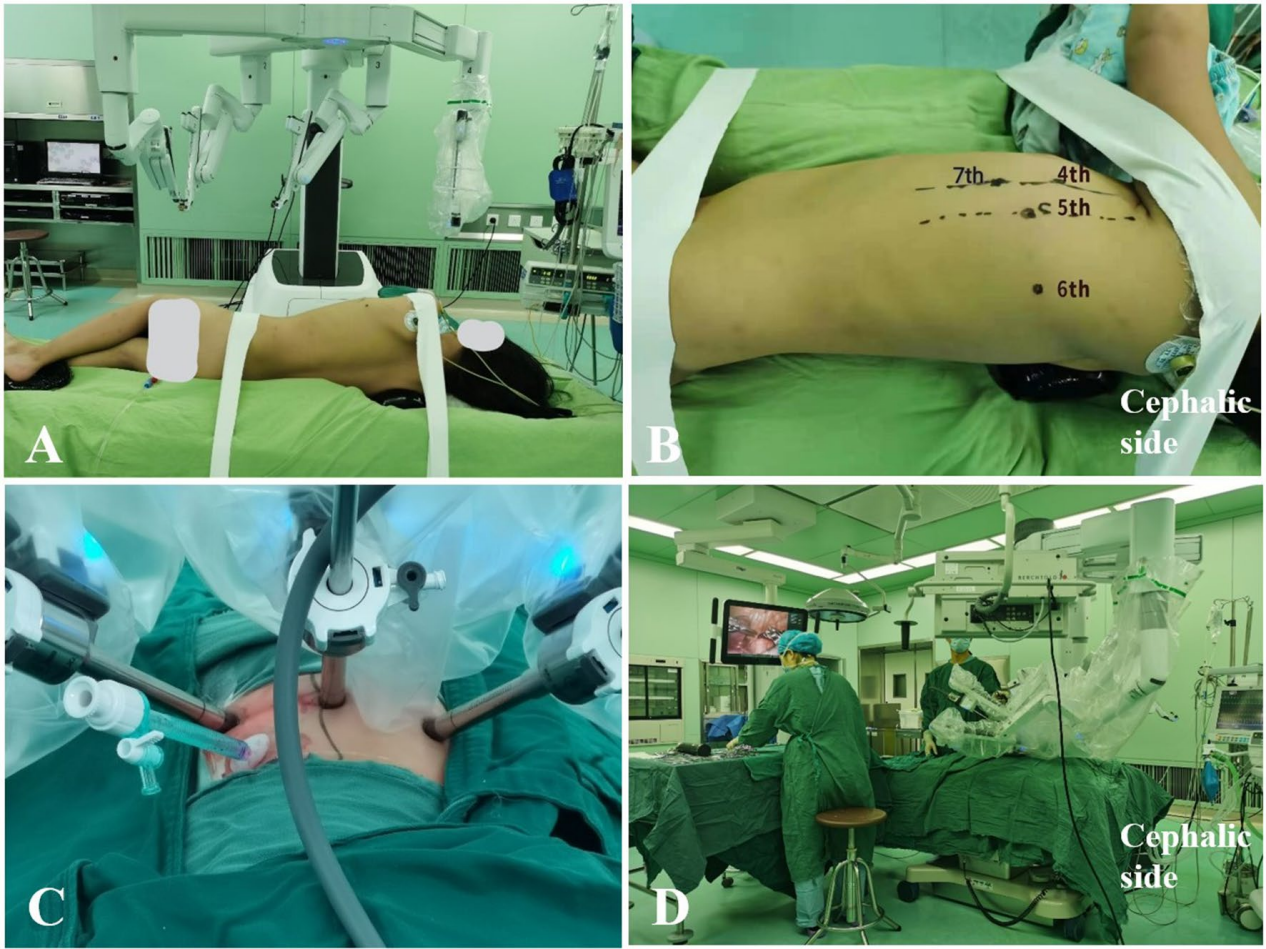
Intraoperative images. **A** The surgical position of the patient. The robot system is on the right side of the operation bed. **B**, **C** The preoperation locations of the trocar foramen and the intraoperative instruments position. **D** Position of the surgical team works and the robot instruments. The robot instruments were on the head side of the patient. The assistant surgeon stands on the right side of the operating bed, near the tail. The nurse is on the left side of the operating bed

**Fig. 2:**
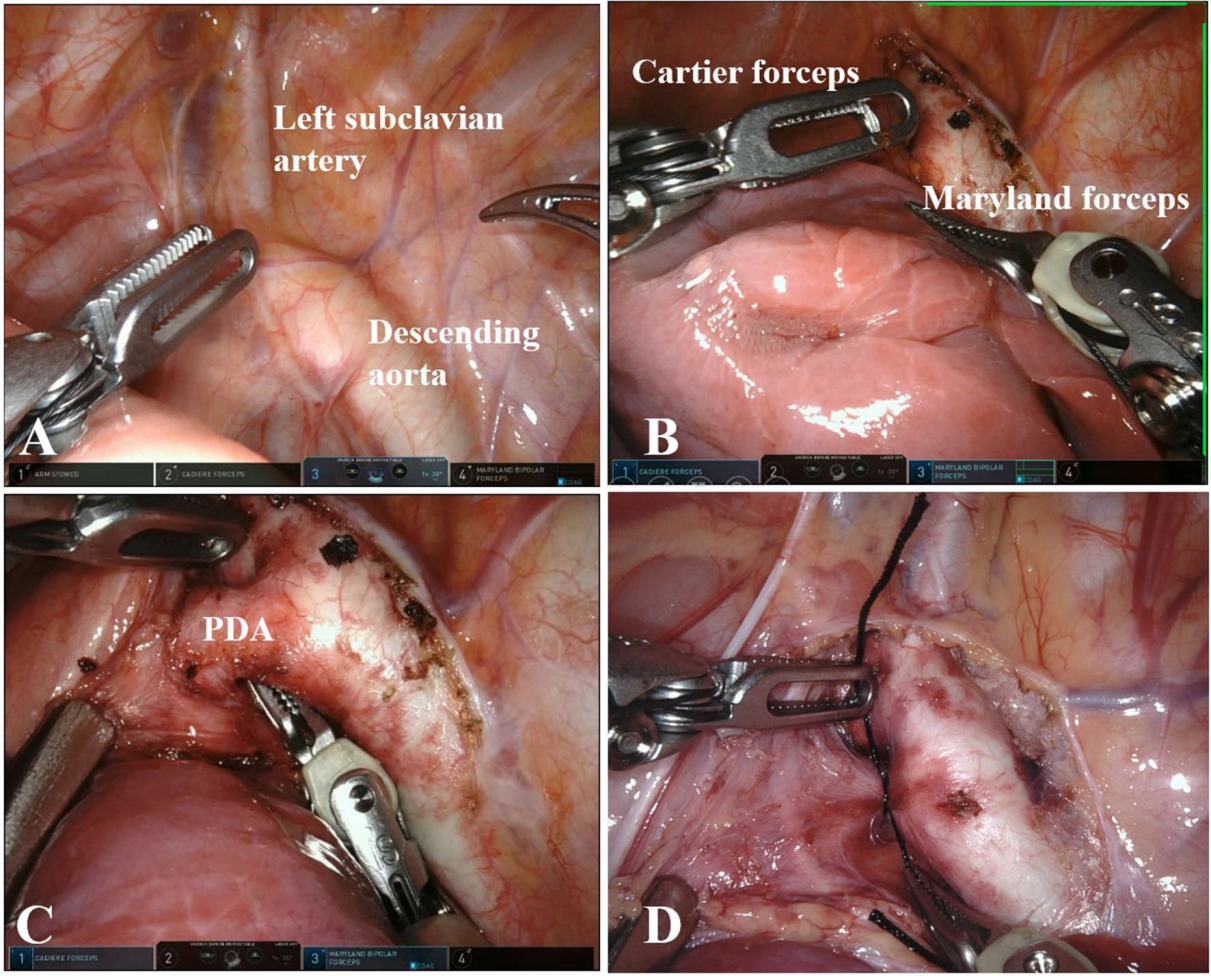
Surgical procedures. **A** The assistant surgeon exposes the PDA by pulling the lobes of the lung. **B** The surgeon opens the mediastinal pleura and aortic adventitia from the left clavicular artery to the lower window of PDA. **C** The surgeon moves Maryland from the back of the lower window to the upper window. **D** The surgeon ligates PDA with silk thread

**Fig. 3:**
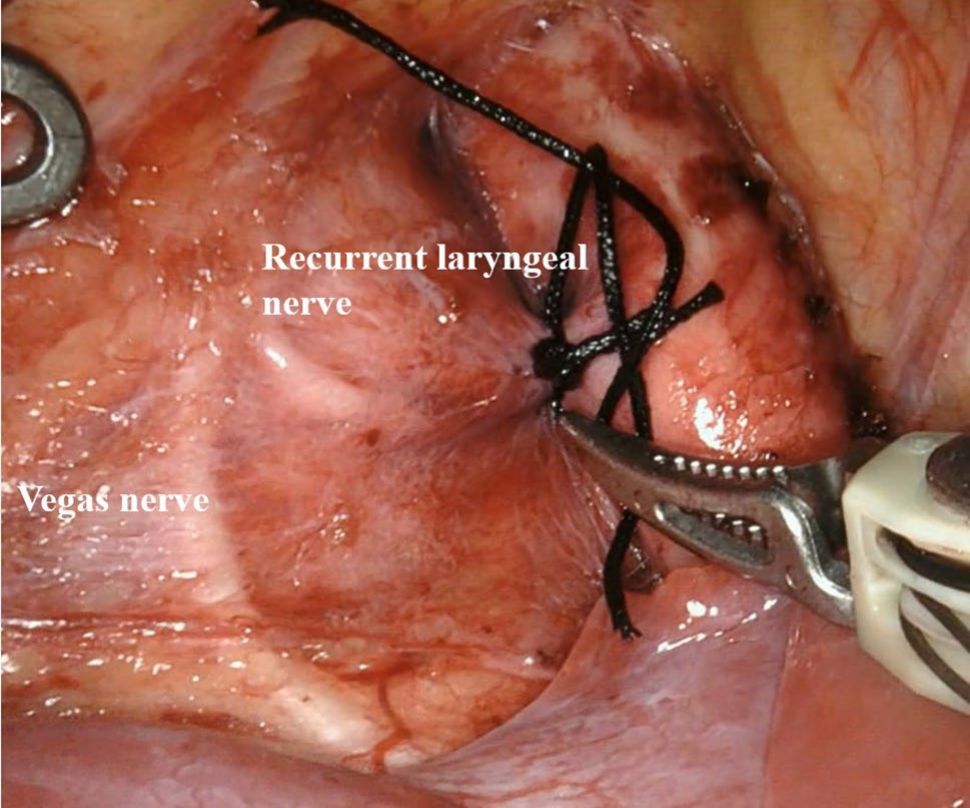
The nerves. The image shows the vagus nerve and its branch as well as the recurrent laryngeal nerve were clamped between the mediastinal pleura and the aortic adventitia

The patients were positioned in the right lateral decubitus position with the right armpit appropriate raised. This would allow easier retraction of the left lung and better visualization of the surgical field. Two cloth tapes with a width of approximate 4 cm were used to fix the anterior superior iliac spine and shoulder seam of the child, respectively. There were three trocars which were placed in the left hemithorax to accommodate the camera and the two robotic manipulators. The observation port was placed in the fifth intercostal space of the midaxillary line. The left operation instrument port was placed in the fourth intercostal space of the anterior axillary line and the right one was in the sixth intercostal space of the mid scapular line, respectively. The fourth-generation Da Vinci robot systems (Intuitive Surgical, Inc. 1266 Kifer Rd, Sunnyvale, CA USA 94086) were placed on the right side of the operating table near the head end. After the artificial pneumothorax pressure was maintained at 4 mmHg, the camera was attached to the robotic cart and the Maryland forceps and Cartier forceps were placed through the left and right trocars, respectively. An auxiliary trocar was inserted in the seventh intercostal space of the anterior axillary line for stretch exposure if necessary. Figure 1 shows the intraoperative images including surgical position of the patient and the robot, location of the trocar foramen and the surgical team works. The assistant pulled the anatomical upper part of the left upper lung to expose the position of the PDA. The two robotic surgical instruments opened the mediastinal pleura and aortic adventitia from the left clavicular artery to the lower window of PDA. The forceps from the auxiliary trocar pulled the outer membrane to expose the artery conduit tissue. The surgeon gently exposed the full view of the PDA, then separated the upper and lower windows bluntly. Subsequently, the surgeon moved Maryland from the back of the lower window to the upper window. Two silk threads were used to ligate PDA when reducing the systolic pressure to approximately 60 mmHg (see Fig. 2). To avoid injuring the vagus nerve and its branch as well as the recurrent laryngeal nerve, these nerves were clamped between the mediastinal pleura and the aortic adventitia, (see Fig. 3). Endotracheal intubation was returned to the main bronchus for bilateral pulmonary ventilation. At the same time, transesophageal echocardiography was performed to confirm the absence of residual ductal leakage. After the pneumothorax was discharged and the lung was expanded, the skin trocar ports were sutured.

**Fig. 4:**
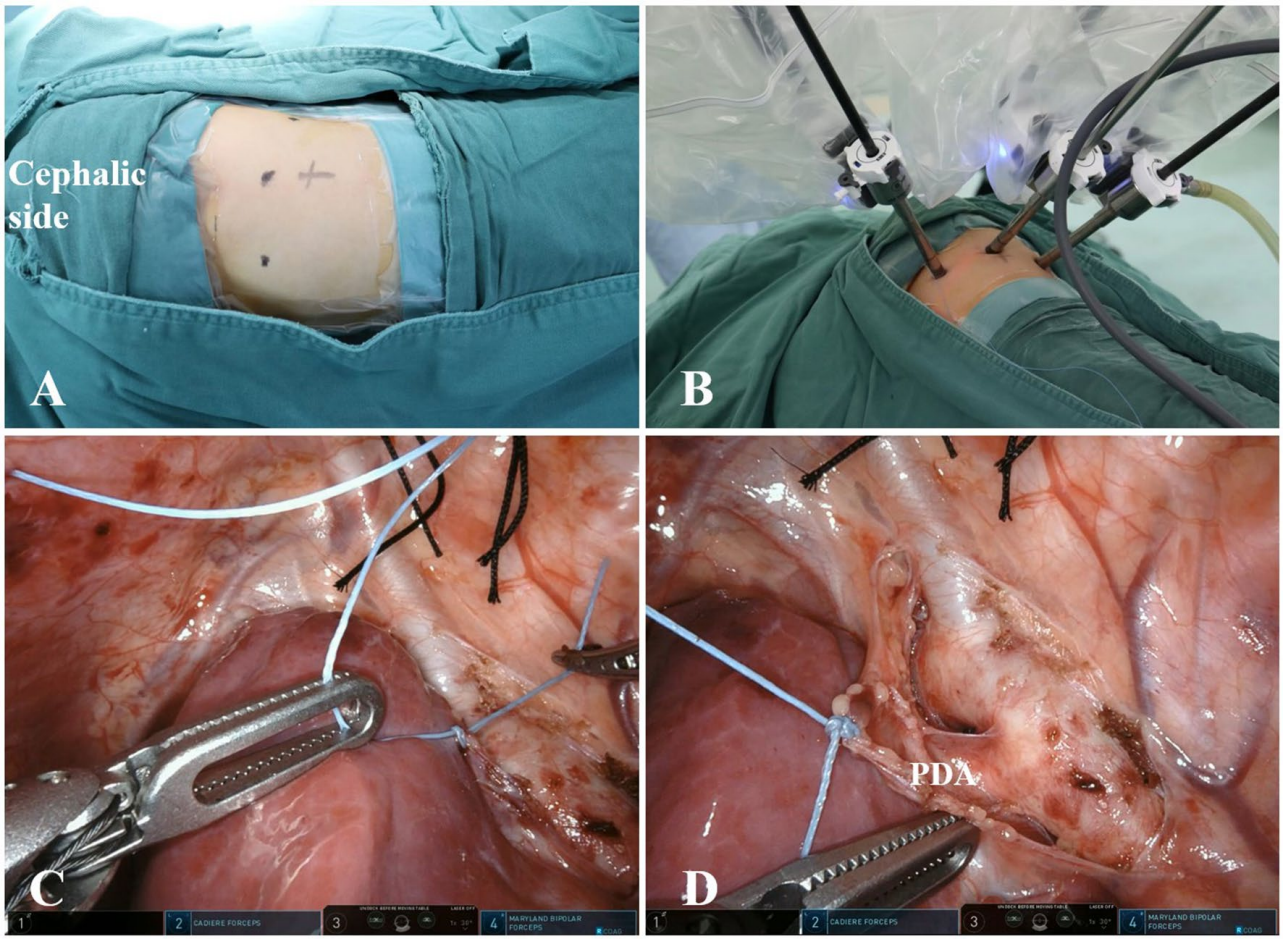
Three-trocar ports method of surgical procedures. **A**, **B** The preoperation locations of the trocar foramen without an auxiliary trocar port and the intraoperative instruments position. **C** A silk thread was sewn on the detached vascular adventitia and knotted. **D** The silk thread was pulled to expose the PDA

With the operation technology becoming more proficient gradually, we performed for only three-trocar ports without an auxiliary trocar in the patients with enough space in the thoracic cavity to expose the robot’s left and right manipulator arms. In these patients, a silk thread passing through the chest wall through the second intercostal space of the anterior axillary line. The silk thread was sewn on the detached vascular adventitia and knotted to instead of the auxiliary instrument. The assistant surgeon pulled the silk thread with appropriate force to expose the PDA. Figure 4 shows the relative position of the three robot instrument arms on the patient, as well as the vision surgical field.

## Results

**Fig. 5:**
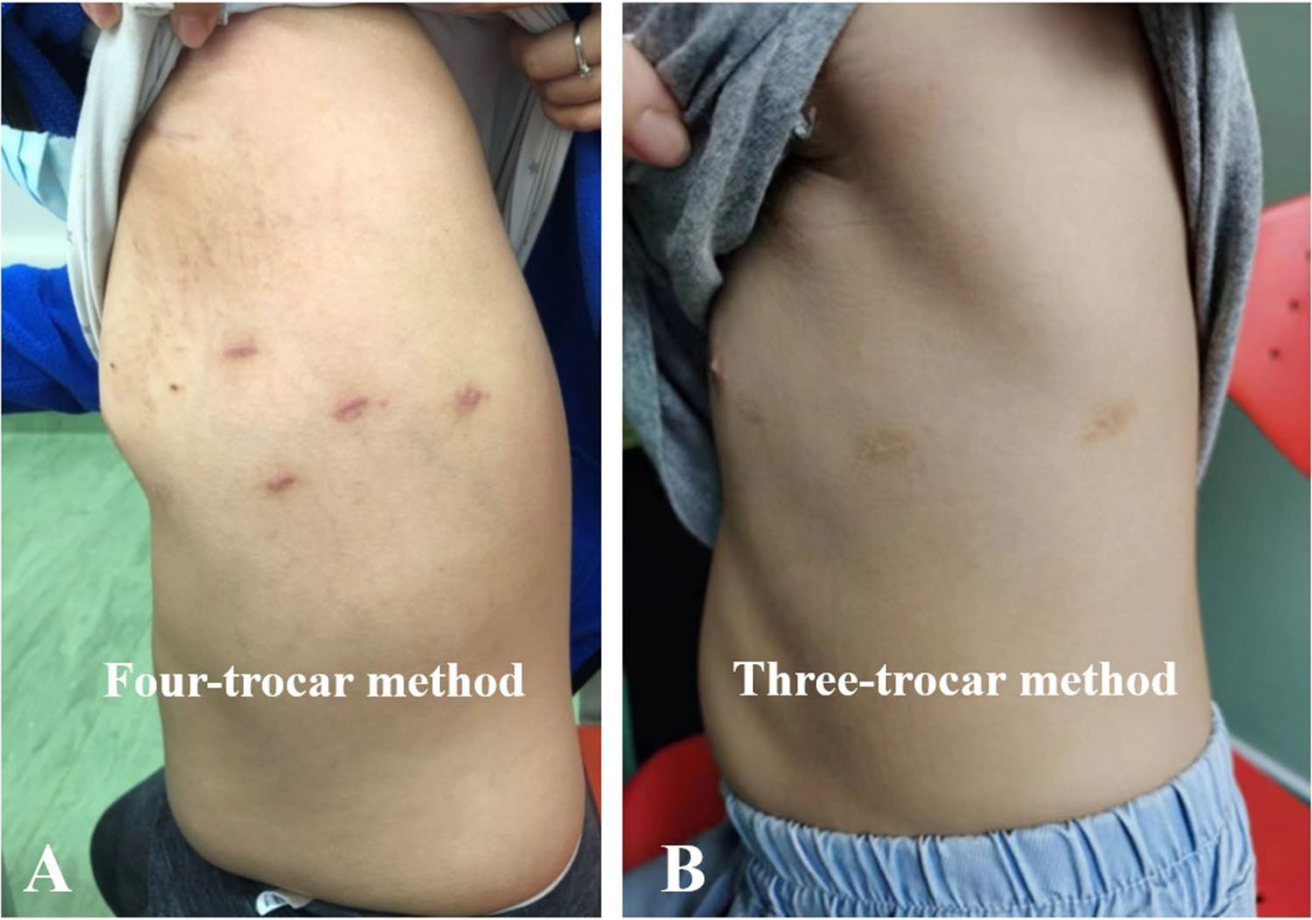
Skin incision 1 month later. The images show the skin incision in **A** the four-trocar method procedure and that in **B** the three-trocar method procedure

The clinical data of all patients are shown in Table 1. There was no significant difference in age, weight, or PDA diameter data of the patients between the robotic surgical group and the transcatheter closure group. The ages ranged from 6 months to 12 years with a median age of 2.5 years, and the weight ranged from 6.6 kg (kg) to 51.6 kg with a median weight of 12.5 kg in the robotic surgical group. In addition, the PDA diameter was between 0.15 and 0.71 cm, with a median diameter of 0.31 cm in the robotic surgical group. All 106 cases underwent surgery successfully with the assistance of the Da Vinci robot. No thoracotomy surgery was done. Forty cases were performed for only three-trocar ports without an auxiliary hole. Besides the setting up and docking time, the operation time was 15–84 min with an average of 39.43 min. The intraoperative blood loss was 2–3 ml. No thoracic drainage tube was used. The average hospitalization cost was US$8180, more than the cost for transcatheter closure (*P* < 0.05). The hospitalization fee consists of bed fee, perioperative examination fee, anesthesia fee, operation fee and the surgical consumables. The high price of the robotic surgical consumables was the real reasons for more hospitalization cost in the robotic surgical patients. No abnormality was found in echocardiography or chest X-ray before discharge. We also successfully performed day surgery in 35 cases. The incision healed well 1 month after discharge (see Fig. 5).

**Table 1:** Robotic surgical and transcatheter closure PDA data of children

	Robotic surgical data of children	Transcatheter closure PDA data of children	*P*
Cases (*n*)	106	225	
Age (year)	3.1 ± 2.1	3.0 ± 2.0	> 0.05
Body weight (kg)	15.8 ± 8.4	15.1 ± 10.2	> 0.05
PDA diameter (cm)	0.36 ± 0.13	0.34 ± 0.14	> 0.05
Operation time (minute)	39.4 ± 17.9	35.6 ± 18.4	< 0.05
Blood loss (ml)	2.0 ± 0.1	5.2 ± 2.3	< 0.05
Postoperative hospital stay (day)	1.1 ± 0.2	2.2 ± 0.5	< 0.05
Cost (US$)	8180 ± 384	5076 ± 406	< 0.05

One patient had residual ductus arteriosus leakage of 0.12 mm detected by echocardiography 1 month after the operation. The PDA diameter of this patient was 0.56 cm before the operation. No residual leakage was found in the intraoperative and postoperative ultrasonography before discharge. This residual leakage reduced to less than 1 mm 6 months after the operation and vanished 9 months after discharge. One patient had hoarseness and choking cough after surgery, which improved after 1 months and recovered 3 months of follow-up. We have performed follow-ups for all patients for 1–20 months with a median of 12 months. No other midterm complications were observed during the follow-up time.

## Discussion

As a normal part of fetal circulation, the ductus arteriosus connects the aorta and the pulmonary artery to shunt blood away from the nonfunctioning lungs. Typically, this conduit will close on its own within the first few days of birth. In some infants, especially neonates, the ductus arteriosus remains open. The incidence rate of PDA in term infants is 0.057% [6]. This persistent shunt, allowing blood to flow from the aorta into the pulmonary artery, can create high blood pressure in the pulmonary artery. The result of increased blood flow to the lungs and decreased blood flow to the rest of the body is lung injury and systemic effects such as cerebral hemorrhage, necrotizing enterocolitis and Eisenmenger’s syndrome. Thus, intervention is recommended if PDA is present. There are several options for PDA treatment; however, each method has advantages and disadvantages. There are many problems in the ligation or suture of PDA through posterolateral posterior incision, such as large trauma, slow recovery and scar formation [7]. This type of ligation is still suitable for all kinds of PDA, including large PDA and window PDA. Drug therapy is only suitable for the treatment of PDA in premature infants [8]. Interventional PDA occlusion has the disadvantages of radioactivity, the long-term existence of occluder in body, postoperative hemolysis, and limited MRI examination [9–12]. These were also the main reasons why our patients chosen robotic surgical. Thoracoscopic PDA ligation has some disadvantages, such as conversion to thoracotomy, unclear intraoperative visual field, difficulty in detaching and ligating PDA, and intercostal pain [13–16].

The present study demonstrated that endoscopic PDA closure with robotically assisted instrumentation was technically feasible in children. Furthermore, dissection of the aorta, subclavian artery and ductus were performed easily and safely using EndoWrist instruments, including an articulated grasper, a hook-up cautery on a low energy setting, and articulated scissors, with no laryngeal nerve injuries or hemorrhage. Enhanced intracorporeal dexterity, optimized hand-eye alignment, and tremor filtering made tissue handling and dissection easy and accurate. Most importantly, there is stationary pivot point at the chest wall. This significantly reduced postoperative pain due to intercostal muscle traction and rib compression. Since there was no tension in the intercostal space and no thoracic drainage tube, there was no compression or traction on the intercostal nerve and subcutaneous tissue, inducing little pain and quick recovery. The patients could get out of bed 6 h after the operation and leave the hospital on the first day after the operation. At the same time, there was no need to use antibiotics during the perioperative period.

Compared with the traditional endoscope, the fourth generation of robot surgical systems has the following advantages: clear and accurate three-dimensional vision, intelligent actions, motion correction and jitter filtering. The surgeon does not need to go to the operating table to avoid crowding between the surgeon and the assistant or blocking the surgical field of vision. In our experience, with a small number of cases, patients aged more than 6 months and weighing more than 5 kg were chosen. In fact, patient younger than 6 months had been reported to be treated using robotic surgical technique [17]. Moreover, the diameter of the PDA was less than 0.8 cm, which was considered safe for ligation even for a window-type duct. To date, the fourth generation of robot surgery systems (Da Vinci robot) has been widely used in adult urology, thoracic surgery, obstetrics and gynecology, general surgery, cardiac surgery, and so on [18, 19]. With the development of minimally invasive technology, the Da Vinci robot is even used in head and neck surgery [20]. However, application of the Da Vinci robot in pediatric surgery is relatively restrictive because children’s body cavity space is narrow and traditional procedures are not applicable. The progress of endoscopic surgery has gradually solved this problem, but there are still disadvantages for accurate operation. The Da Vinci robot can perform a perfect operation in a limited space, can reduce surgical injury, can improve the curative effect and can minimize pain in children. Therefore, the Da Vinci robot has advantages in the application of pediatric surgery. At present, the robot system is mainly used in pediatric urology. The history of robot application in pediatric cardiac surgery is relatively recent. El Bret first reported in 2002 that PDA was cut and sutured with robot assistance [21]. PDA ligation through a robot system was also reported in 2005 by Yoshihiro Suematsu in Boston Children’s Hospital [5]. At present, there is no published report about the robot system used in pediatric cardiac surgery in China. The number of PDA ligation by Da Vinci robots is small. The indications and contraindications are relative. With the increasing number of patients, the indications of robotic PDA ligation may expand.

The selection of the position of the trocar placement was of very importance in the operation, owing to the poor position of the trocar inducing poor vision, difficulty in operation, and relative resistance between the manipulator’s arms. In this group, the 4 trocar method effectively avoided interference from the visual field of the left upper lobe, and the method was safe for separating and ligating the PDA. In the early application period, the distance between the mirror trocar and the right main manipulators was too short in one case, resulting in confrontation between the two instruments. Thus, at that time, the operation was extremely difficult, and 85 min was required to complete the surgery. With the accumulation of experience, we also performed a 3-port operation without an auxiliary trocar. It was proved to be feasible. Due to lack of experience in the early stage of this group, we selected children aged > 2 years and weighing > 10 kg for surgical treatment. With the accumulation of case data, infants aged < 2 years or even infants can be considered for Da Vinci robot surgery. Except PDA procedure, the treatment of congenital heart diseases in children with the assistance of the Da Vinci robot is limited because of age and weight requirements for the establishment of cardiopulmonary bypass. In China, it is used only in the surgical treatment of atrial septal defects and in ventricular septal defects in older children [22]. With the increasing number of patients, the indications of robotic PDA ligation may expand.

The magnification of the visual field can be adjusted according to the need for the intraoperative condition and the habit of the operator. After separating the upper and lower windows of the PDA, the vagus nerve and its recurrent laryngeal branch can be displayed and avoid to be injury during ligation. One patient had clinical symptoms of laryngeal nerve injury, such as hoarseness and choking cough. For this patient, it was thought that the recurrent laryngeal nerve injury duo to the mediastinal pleura and aortic adventitia were not separated effectively. Therefore, to avoid nerve injury, we separated the two layers as much as possible. It is better to sandwich the vagus nerve and its recurrent laryngeal branch between the two layers. The robot Endowrist function can effectively, safely and easily detach the posterior wall of PDA. In addition, a high-definition field of vision makes deep ligation quick and safe. One patient was found to have residual ductus arteriosus by echocardiography 1 month after the operation. Considering the large size of the ductus arteriosus, the ligation of the ductus arteriosus was not tight enough to result in residual ductus arteriosus. In the later stage, we ligated large PDA three times to prevent residual leakage. Meanwhile, esophageal ultrasound and transthoracic ultrasound were also needed to evaluate residual leakage.

### Limitations

In our experience, robot-assisted PDA ligation also has the following technical restrictions. First, there is no force feedback during the operation of the manipulator, so special care should be taken in separating the posterior wall of the ductus arteriosus through the line of sight and the position sense of the instrument. When ligating the arterial duct, it is necessary to observe the tension of the silk thread closely to prevent the silk thread from breaking due to excessive force. Second, this technic requires a surgeon with at least 10 years of experience to ligate the PDA in thoracotomy in case of a rupture of arterial duct tissue. In this group, the chief surgeon had more than 10 years of experience in cardiac surgery, and there were no cases of massive bleeding, silk thread breakage, or conversion to thoracotomy. Fortunately, a previous study has commented on the learning curve for robotic surgery being shorter than that for endoscopic surgery [23]. In addition, it is reported that the presence of wristed instrumentation, tremor abolition and motion scaling enhanced dexterity by nearly 50% as compared with endoscopic surgery, and three-dimensional vision enhanced dexterity by a further 10–15% in addition to the 93% reduction in skills based errors [24]. Therefore, it is believed that robotic surgical technique would been more easer to master and accept. In addition, the average cost of robot-assisted endoscopic treatment of PDA surgery is US$8180. Since this surgical procedure has not entered medical insurance reimbursement, although the cost is not very expensive in most eastern China family, it may be considered a burden for families with relatively low economic levels. It is just an alternative to traditional approaches for some families with special requirements. It offers patients psychological and social satisfaction and quality of life without occluder device in body. We had tried our best to optimize the operation procedures. The cost of the surgery was reduced, to some extent, due to the cancelation auxiliary trocar. We hope that with the improvement of instrument technology, the cost of this operation will be reduced rapidly and applicable to all families. Lastly, PDA surgery through robotic systems has been performed based on a small number of patients in a single center. There is required multicenter experience in the future.

## Conclusion

At present, our initial experience demonstrates that robotically assisted PDA closure in children is feasible and safe procedure with good prospects. Owing to afford the improved visualization and dexterous manipulation, The Da Vinci robotic system has the advantages of less trauma, safety, rapid recovery and short hospital stay in the treatment of PDA in children. It should be considered as an option in PDA treatment.

## Contribution 

There are no other papers, with large number samples, since 2005 reported Da Vinci robot-assisted endoscopic technique used for PDA treatment. Da Vinci robot technique has been applied in all fields in the last 15 years, as well as in cardiac surgery. However, in the field of pediatric heart surgery, little progress has been made. Our manuscript analyzed the clinical data and tries to sum up more experience of robot technique for PDA treatment. As we known at present, this is the largest sample of analysis the application of robotic surgery in PDA. We demonstrate that robotically assisted PDA closure in children is feasible and safe procedure with good prospects. Owing to afford the improved visualization and dexterous manipulation, the Da Vinci robotic system has the advantages of less trauma, safety, rapid recovery and short hospital stay in the treatment of PDA in children. It should be considered as an option in PDA treatment. 

## Data Availability

The original contributions presented in the study are included in the article/supplementary material, and further inquiries can be directed to the corresponding authors.
